# Validating a web application’s use of genetic distance to determine helminth species boundaries and aid in identification

**DOI:** 10.1186/s12859-025-06098-0

**Published:** 2025-03-18

**Authors:** Abigail Hui En Chan, Urusa Thaenkham, Tanaphum Wichaita, Sompob Saralamba

**Affiliations:** 1 Department of Helminthology, Faculty of Tropical Medicine, Mahidol University, Bangkok, Thailand; 2 Mahidol Oxford Tropical Medicine Research Unit, Faculty of Tropical Medicine, Mahidol University, Bangkok, Thailand

**Keywords:** Application, Helminth, Genetic marker, Species boundaries, K-mean

## Abstract

**Background:**

Parasitic helminths exhibit significant diversity, complicating both morphological and molecular species identification. Moreover, no helminth-specific tool is currently available to aid in species identification of helminths using molecular data. To address this, we developed and validated a straightforward, user-friendly application named Applying Taxonomic Boundaries for Species Identification of Helminths (ABIapp) using R and the Shiny framework. Serving as a preliminary step in species identification, ABIapp is designed to assist in visualizing taxonomic boundaries for nematodes, trematodes, and cestodes. ABIapp employs a database of genetic distance cut-offs determined by the K-means algorithm to establish taxonomic boundaries for ten genetic markers. Validation of ABIapp was performed both in silico and with actual specimens to determine its classification accuracy. The in silico validation involved 591 genetic distances sourced from 117 publications, while the validation with actual specimens utilized ten specimens. ABIapp’s accuracy was also compared with other online platforms to ensure its robustness to assist in helminth identification.

**Results:**

ABIapp achieved an overall classification accuracy of 76% for in silico validation and 75% for actual specimens. Additionally, compared to other platforms, the classification accuracy of ABIapp was superior, proving its effectiveness to determine helminth taxonomic boundaries. With its user-friendly interface, minimal data input requirements, and precise classification capabilities, ABIapp offers multiple benefits for helminth researchers and can aid in identification.

**Conclusions:**

Built on a helminth-specific database, ABIapp serves as a pioneering tool for helminth researchers, offering an invaluable resource for determining species boundaries and aiding in species identification of helminths. The availability of ABIapp to the community of helminth researchers may further enhance research in the field of helminthology. To enhance ABIapp’s accuracy and utility, the database will be updated annually.

**Supplementary Information:**

The online version contains supplementary material available at 10.1186/s12859-025-06098-0.

## Background

Parasitic helminths, which comprises of the phyla Nematoda and Platyhelminthes are highly diverse and globally distributed [1, 2]. While estimates of helminth diversity remain controversial due to the small proportion of these organisms described, Carlson et al. (2020) estimated a global total of 100,000 helminth species, with 85–95% still unknown [2]. Factors such as their complex life cycles, ability to switch hosts resulting in rapid adaptive radiation, parasitism across various hosts, and ubiquitous presence in diverse ecological habitats like soil and marine environments contribute to the vast species diversity of helminths [3, 4].

Traditionally, helminth species identification relied on morphological characteristics. However, challenges arise from ambiguous morphological features, phenotypic plasticity from diverse hosts and habitats, technical variations in specimen preparation, and incomplete specimens missing key diagnostic morphological characters [5–7]. The molecular era introduced genetic markers as alternative identification tools. These markers not only expedited molecular-based helminth identification but also allowed for accurate differentiation of previously morphologically indistinguishable species [5–9]. However, despite the benefits of molecular identification, challenges arise due to species complexes and cryptic species [2, 8, 9]. It has been estimated that there are, on average, 2.4 cryptic species per cestode species, 3.1 for trematodes, and 1.2 for nematodes [2]. Genotypic variation complicates species boundary definitions and consensus on species delimitation. The presence of species complexes, species from different geographic localities, and varied hosts may thereby result in increased genotypic variation within a species. Typically, using mitochondrial genes, distinct species exhibit a 5–10% genetic distance [5]. However, a study by Chan et al. (2021) on ten general genetic markers for parasitic helminths highlighted considerable genetic variations and questioned the use of a general genetic distance value to determine whether helminth specimens are conspecific [10]. The ten genetic markers were the nuclear 18S and 28S ribosomal RNA (rRNA) genes, nuclear internal transcribed spacer 1 and 2 (ITS1 and ITS2) regions, the mitochondrial 12S and 16S rRNA genes, the mitochondrial protein-coding genes of cytochrome *c* oxidase subunits 1 and 2 (*COI* and *COII*), cytochrome b (*cytb*), and NADH dehydrogenase subunit 1 (*ND1*).

Various methods, including those based on phylogenetic reconstruction or distance-based calculations, have been employed to determine species boundaries among organisms. Notable among phylogenetic methods are the Bayesian modeling approach, the General Mixed Yule Coalescent (GMYC) and multi-coalescent model approach, and the Poisson tree processes (PTP) model [11–16]. For instance, Pons et al. (2006) applied the GMYC model for beetle speciation [12], while Yang and Rannala (2014) integrated gene trees using multiple loci [15]. However, these models have not been extensively adopted for helminths. On the other hand, distance-based methods like the Automatic Barcode Gap Discovery (ABGD) and Assemble Species by Automatic Partitioning (ASAP) have been explored [17–20], while Chan et al. (2021) also introduced a K-means algorithm-based method to define helminth species boundaries using genetic distances [10]. The K-means method uses clustering to partition datapoints to minimize the within-cluster sum of squares to minimize the pairwise squared deviations of points in the same cluster. The K-means method thus allows for a convenient method to determine cut-off values with a dataset of genetic distances and allows clustering into a pre-defined number of clusters [21, 22]. The cut-off genetic distance values obtained [10] were then input into our application to define helminth species boundaries for each taxonomic hierarchy level per genetic marker per helminth group.

In this paper, we present ABIapp, a user-friendly application designed to make the K-means species boundaries for helminths accessible to a wider audience. ABIapp offers a selection of ten genetic markers, allowing users to choose the most suitable one for their research. The application requires minimal bioinformatic input, making it a convenient and preliminary tool for users to gauge species boundaries for their genetic marker used. The output obtained from ABIapp may eventually provide informed choices that can aid in helminth species identification. To enhance ABIapp’s utility, we expanded the sequence database and updated the estimated K-means cut-off genetic distance values. We then validated ABIapp’s robustness and applicability by assessing its classification accuracy against both previously published genetic distances and actual specimens. Finally, we compared ABIapp’s accuracy with other online platforms like ASAP, ABDG, and PTP. Note that in silico validation focused solely on ABIapp’s classification accuracy without passing judgment on the correctness of the methods. Our efforts have led to the creation of the first validated and accessible application specifically for parasitic helminth (nematodes, trematodes, and cestodes), now accessible to the research community.

## Implementation

### Determination of estimated genetic distances using the K-means algorithm

The database of helminth genetic distances was used to estimate the cut-off genetic distance values through the K-means algorithm. Following the method from Chan et al. (2021), helminth sequences from ten genetic markers (nuclear 18S and 28S rRNA genes, the nuclear ITS1 and ITS2 regions, and the mitochondrial *COI*, *COII*, *cytb*, *ND1*, and 12S and 16S rRNA genes) were extracted from the NCBI database [10]. For mitochondrial genes, full-length sequences were sourced from complete mitochondrial genomes, while full-length or near full-length sequences were selected for nuclear genetic markers. The helminths were categorized into six groups: nematode clade I (Trichocephalida), nematode clade III (Ascaridida and Spirurida), nematode clade V (Strongylida), trematode (Plagiorchiida), trematode (Diplostomida), and cestode, in accordance with the taxonomic classification proposed by Blaxter et al. (1998) and Olson et al. (2003) [23, 24].

In brief, sequence alignments were conducted using ClustalX 2.1 and Bioedit 7.0 for each genetic marker within each helminth group [25, 26]. Subsequently, pairwise genetic distance calculations were carried out in MEGA X to determine genetic distance values for each genetic marker by taxonomic hierarchy within each helminth group [27]. These genetic distance values were then processed in Wolfram Mathematica 12.1 to derive estimated cut-off genetic [28] distance values for each taxonomic level using the unsupervised K-means clustering algorithm. The number of clusters chosen corresponded to the taxonomic levels of the genetic distance values; for instance, four clusters would represent the “species”, “genus”, “family”, and “order” levels. The helminth sequences used, along with the estimated K-means values, are detailed in Additional Tables 1 and 2, respectively. These estimated K-means values were then used as a database for the ABIapp to visualize taxonomic boundaries of the target helminth group of interest and genetic marker.

**Table 1:** Actual specimens used to validate ABIapp

Helminth group	Order/suborder	Family	Genus	Species	Stage	Host	Location
Nematode clade I	Trichocephalida	Trichuridae	*Trichuris*	*Trichuris globulosa*	Adult	Arabian camel	Kuwait
Nematode clade III	Spirurida	Gnathostomatidae	*Gnathostoma*	*Gnathostoma* sp.	Larva	Asian eel	Thailand
	Spirurida	Gnathostomatidae	*Tanqua*	*Tanqua* sp.	Adult	Snake	Thailand
Nematode clade V	Strongylida	Strongylidae	*Oesophagostomum*	*Oesophagostomum stephanostomum*	Adult	Chimpanzee	Uganda
Trematode Plagiorchiida	Pronocephalata	Gastrothylacidae	*Gastrothylax*	*Gastrothylax* sp.	Adult	Cattle	Thailand
	Xiphidata	Dicrocoellidae	*Eurytrema*	*Eurytrema* sp.	Adult	Cattle	Thailand
	Opisthorchiata	Heterophyidae	*Centrocestus*	*Centrocestus formosanus*	Adult	Hamster	Thailand
	Opisthorchiata	Heterophyidae	*Stallantchasmus*	*Stallantchasmus falcatus*	Adult	NA	Thailand
Trematode Diplostomida	Diplostomida	Clinostomidae	*Clinostomum*	*Clinostomum* sp.	Adult	Asian eel	Thailand
Cestode	Cyclophyllidea	Anoplocephalidae	*Bertiella*	*Bertiella* sp.	Adult	Chimpanzee	Uganda

NA indicates no information available

**Table 2:** Primers used for ABIapp validation with actual specimens

Specimen	Genetic marker	Primer (5′-3′)	Reference
*Trichuris* sp.	*COI*	HC02198F: TTTTTTGGGCATCCTGAGGTTTAT	[32]
		CORA: ACYACATAGTAGGTRTCATG	
	12S	12S-C1-F: GTGCCAGCTAYCGCGGTTA	[33]
		12S-C1-R: GRTGACGGGCRATATGTG	
	16S	16S-C1-F: ACGAGAAGACCCTRGRAAYT	[33]
		16S-C1-R: GRTYTAAACTCAAATCACG	
	18S	1096F: GGTAATTCTGGAGCTAATAC	[34]
		1912R: TTTACGGTCAGAACTAGGG	
		1813F: CTGCGTGAGAGGTGAAAT	
		2646R: GCTACCTTGTTACGACTTTT	
*Gnathostoma* sp., *Tanqua* sp., and *Oesophagostomum stephanostomum*	*COI*	JB3: TTTTTTGGGCATCCTGAGGTTTAT	[35]
		JB4.5: TAAAGAAAGAACATAATGAAAATG	
	12S	12S-C345-F: GTWCCAGAATAATCGGMTA	[33]
		12S-C345-R: ATTGAYGGATGRTTTGTRC	
	16S	16S-C345-F: AAGATAAGTCTTYGGAARYT	[33]
		16S-C345-R: GAAYTAAACTAATATCAMG	
	18S	1096F + 1912R, 1813F + 2646R	[34]
Trematode and cestode	*COI*	JB3 + JB4.5	[35]
	12S	Tre12S-F: GTGCCAGCADYYGCGGTTA	[37]
		Tre12S-R: AGCAGCAYATHGACCTG	
		Ces12SF: GTGCCAGCATCYGCGGTTA	[36]
		Ces12SR: GGTGACGGGCGGTGTGTAC	
	16S	CesTre16S-F: GTGYDAAGGTAGSATAAT	[37]
		CesTre16S-R: CCGGTYTYAACTCARCTCAT	
	18S	Cfor: ATGGCTCATTAAATCAGCTAT	[38]
		Arev: TGCTTTGAGCACTCAAATTTG	

### ABIapp development and workflow

**Fig. 1:**
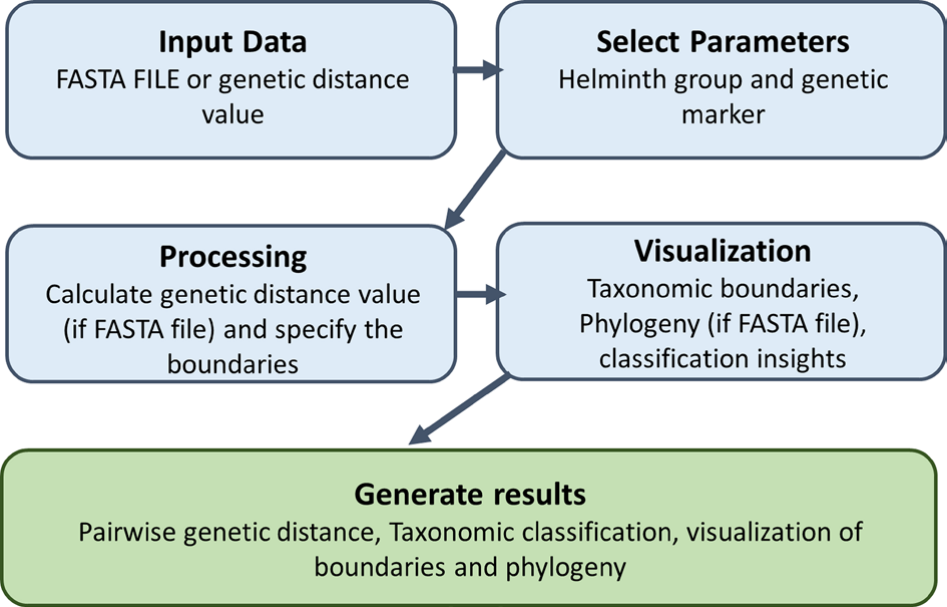
Workflow of ABIapp for genetic distance-based helminth taxonomic identification. ABIapp begins with data input as a FASTA file or genetic distance value. Users select the helminth group and genetic marker, and if a FASTA file is used, pairwise genetic distances are calculated. Outputs include taxonomic boundaries, genetic distance ranges, a phylogenetic tree (if applicable), and classification insights, providing a comprehensive analysis of the queried taxa

The ABIapp was developed using the R programming language (version 4.3.1) [29], and the Shiny web application framework (version 1.9.1) [30] was employed to create an interactive and user-friendly interface. The source code for ABIapp is available at https://github.com/slphyx/ABIApp. ABIapp utilizes a database of helminth genetic distances to establish taxonomic boundaries through the K-means machine learning algorithm, as previously compiled by Chan et al. [10]. The workflow of the application is illustrated in Fig. 1.

**Fig. 2:**
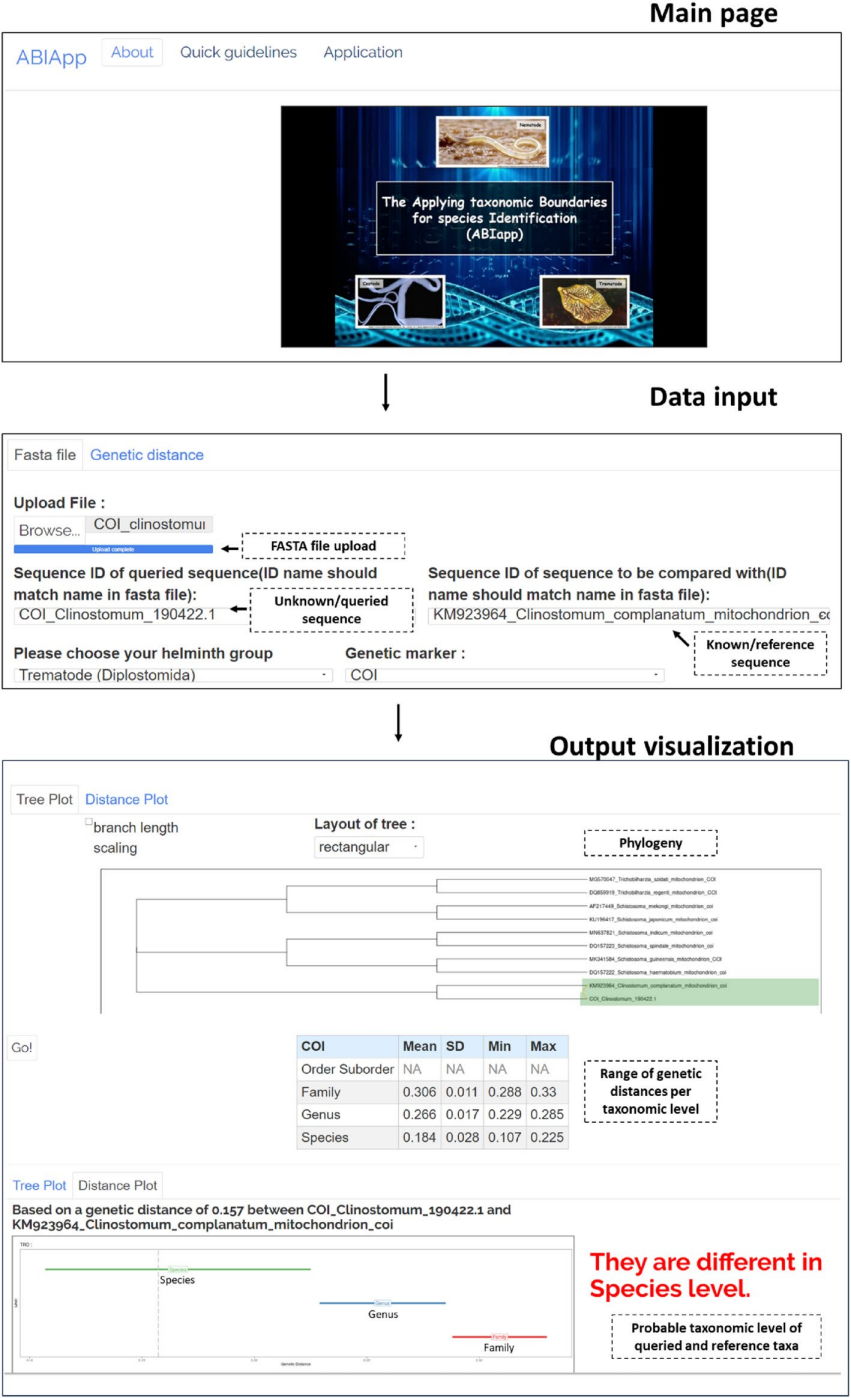
The ABIapp interface displays the main page, input data options, and a sample of the generated results. Based on the queried and reference sequences, the results revealed that the sequences belong to different species, with a genetic distance of 0.157 between them using the *COI* gene. The genetic distance value obtained indicates that it falls within the range of the cut-off distance (0.107–0.225) between species

Designed as a user-friendly application, ABIapp allows users to input either a FASTA file (after multiple sequence alignment) or a genetic distance value derived from pairwise sequence analysis (comparing the queried sequence with reference taxa). The FASTA file should not be more than 2 MB in size. Subsequently, users choose their desired helminth group (nematodes, trematodes, and cestodes) and genetic marker. If a FASTA file is provided, users must specify the identity names of the queried (unknown) and reference (known species based on the sequence in the FASTA file) taxa to compute a pairwise genetic distance value for comparison. The output displays the calculated pairwise genetic distance value (using the *p*-distance model) between the queried and reference taxa, a visual representation of taxonomic boundaries, a table detailing genetic distance ranges for each taxonomic hierarchy level, and a neighbor-joining phylogenetic tree. However, no phylogenetic tree is generated if a genetic distance value is directly inputted. The results from ABIapp can indicate 1) the probable classification of the queried genetic distance based on taxonomic hierarchy, 2) the range of expected genetic distances for the chosen genetic marker, 3) a tentative conclusion on whether the analyzed specimen belongs to the same species as the reference taxa, and 4) a phylogenetic representation using the neighbor-joining method. Figure 2 illustrates the ABIapp interface along with an example of the generated results.

Before using ABIapp, it is recommended to perform morphological identification of the helminth in question to have an idea of the order or family to which the queried specimen might belong. Subsequently, genetic sequence information from one of the ten genetic markers used in ABIapp should be obtained. The general steps for multiple sequence analysis should be performed, and either the genetic distance or the aligned FASTA file can be input into ABIapp. The taxa chosen as references should belong to the helminth group of the queried taxon (e.g., if the queried taxon is suspected to be *Trichuris* species, the taxa chosen for comparison should be within the order Trichocephalida).

ABIapp can be accessed at https://moru.shinyapps.io/ABIapp/ and is also available as an R package (https://github.com/slphyx/ABIApp). A comprehensive step-by-step guide and additional information are provided on the webpage.

### ABIapp validation

To assess the reliability and accuracy of ABIapp in classifying helminth genetic distances, we conducted a two-pronged validation process: in silico validation using previously published genetic distance data and validation with actual helminth specimens. The in silico validation focused on testing the app’s predictive performance with curated datasets, while the specimen validation involved real-world application using archived helminth samples. These complementary approaches ensure ABIapp’s robustness across different datasets and practical scenarios.

#### In silico validation

For the in silico validation, we mined available helminth genetic distances from publications and input them into ABIapp to evaluate its classification accuracy. The classification accuracy was defined as the proportion of ‘correct’ and ‘incorrect’ results as predicted by ABIapp. This determination was grounded in the genetic distance and prior classification derived from the published data. For instance, a “correct” species-level classification means that ABIapp accurately deduced the genetic distance value at that level. Conversely, an “incorrect” classification indicates ABIapp’s failure to correctly ascertain the genetic distance at the species level, as detailed in Additional Table 3. For publication selection, the criteria guiding selection were: 1) the inclusion of genetic distances not previously integrated into the ABIapp database and 2) the exclusion of publications devoid of phylogenetic analysis. For each genetic marker and helminth group, taxonomic information and genetic distances were compiled, noting the taxonomic hierarchy to which the genetic distance pertained (within species, species, or genus). We prioritized these taxonomic levels as they frequently serve in molecular identification. Efforts were made to source genetic distance data spanning all ten genetic markers and every helminth group. Nevertheless, data on certain genetic markers were sparse due to the limited number of relevant publications. A comprehensive list of referenced publications can be found in Additional Table 4.

Subsequently, we fed the compiled genetic distances into ABIapp to gauge its ability to accurately classify the genetic distance value based on the taxonomic hierarchy classification level as stated in the publication. If the genetic distances sourced spanned a value range, both the minimum and maximum values were input. In instances where the genetic distance fell into the middle range between taxonomic levels, the classification was excluded as the data is uninformative to determine the app’s classification accuracy. Using ABIapp’s predicted outcomes compared against the actual data, a confusion matrix was crafted for each genetic marker per helminth group, facilitating the calculation of classification accuracy and error rate [31] (see Additional Fig. 1). Classification accuracy is computed as:

1 $$Accuracy=\frac{True Positive+True Negative}{True Positive+True Negative+False Positive+False Negative}$$

Meanwhile, the error rate is:

2 $$Error = 1{-}Accuracy$$

A true positive is a situation where both the prediction and actual result concur as ‘yes’, whereas a true negative is when both align as ‘no’. A false negative arises when the prediction is ‘yes’ but the actual result contradicts as ‘no’. Conversely, a false positive emerges when the prediction is ‘no’ but the actual outcome is ‘yes’.

#### Validation with actual specimens

We used representative helminth specimens, previously archived at the Department of Helminthology, Faculty of Tropical Medicine, Mahidol University in Bangkok, to validate ABIapp. We selected specimens comprising representatives of each of the six helminth groups for molecular analysis. The specimens were previously morphologically identified and kept in 70% ethanol as archived specimens. The criteria for specimen selection were either 1) unable to identify them at the species level or 2) uncertain about the accuracy of their morphological species identification. Details about the selected specimens can be found in Table 1.

For the molecular analysis, each helminth specimen was carefully placed into a 1.5-ml microcentrifuge tube and rigorously washed with sterile distilled water. From larger specimens, we excised a small section for DNA extraction while preserving the remaining specimen in 70% ethanol as a reference. In the case of smaller specimens, we used the entire specimen. We then subjected the specimens to tissue homogenization using silica beads in lysis buffer with a TissueLyser LT (Qiagen, Hilden, Germany). We extracted the total genomic DNA from each sample using the DNeasy® Blood & Tissue kit (Qiagen, Hilden, Germany), following the provided manufacturer’s instructions.

For our molecular analysis, we chose the mitochondrial 12S and 16S rRNA, *COI*, and the nuclear 18S rRNA genes as indicative genetic markers. The rationale behind selecting these four genetic markers is the availability of primers that target a wide range of species across nematodes, trematodes, and cestodes. The primers utilized for each specimen are listed in Table 2. PCR was conducted in a final volume of 30 µl, comprising 15 µl of 2X i-TaqTM mastermix (iNtRON Biotechnology, Gyeonggi, South Korea), 10 µM to 50 µM of each primer, and the template DNA. We adhered to the thermocycling conditions specified in the publications introducing these primers [32–38]. We visualized amplicons on a 1% agarose gel stained with RedSafe® (Thomas Scientific, New Jersey, USA). After confirming the amplicons, we purified them using the Geneaid PCR purification kit (Geneaid Biotech Ltd., Taiwan, China). A commercial company, Macrogen (Seoul, South Korea), undertook the sequencing on an automated Sanger sequencer.

We examined the electropherograms of the sequences using Bioedit 7.0 and entered the sequences into NCBI-BLAST to identify potentially similar species [25]. We aligned multiple sequences within the same family or genus using ClustalX 2.1 [26]. Subsequently, the FASTA file containing aligned sequences was uploaded to ABIapp to test its accuracy for the tested helminth specimens. The classification accuracy for ABIapp was then calculated as the proportion of ‘correct’ predictions obtained.

#### Comparison with other online platforms

Using the sequences derived from the 10 species, we entered the genetic information into three online platforms to evaluate their classification accuracy against that of ABIapp. These platforms include ASAP (https://bioinfo.mnhn.fr/abi/public/asap/) [19], ABDG (https://bioinfo.mnhn.fr/abi/public/abgd/abgdweb.html) [20], and bPTP (https://species.h-its.org/) [16]. Similarly, the proportion of ‘correct’ predictions were obtained per platform, and the accuracy from each platform was then compared with the classification accuracy achieved by ABIapp.

**Fig. 3:**
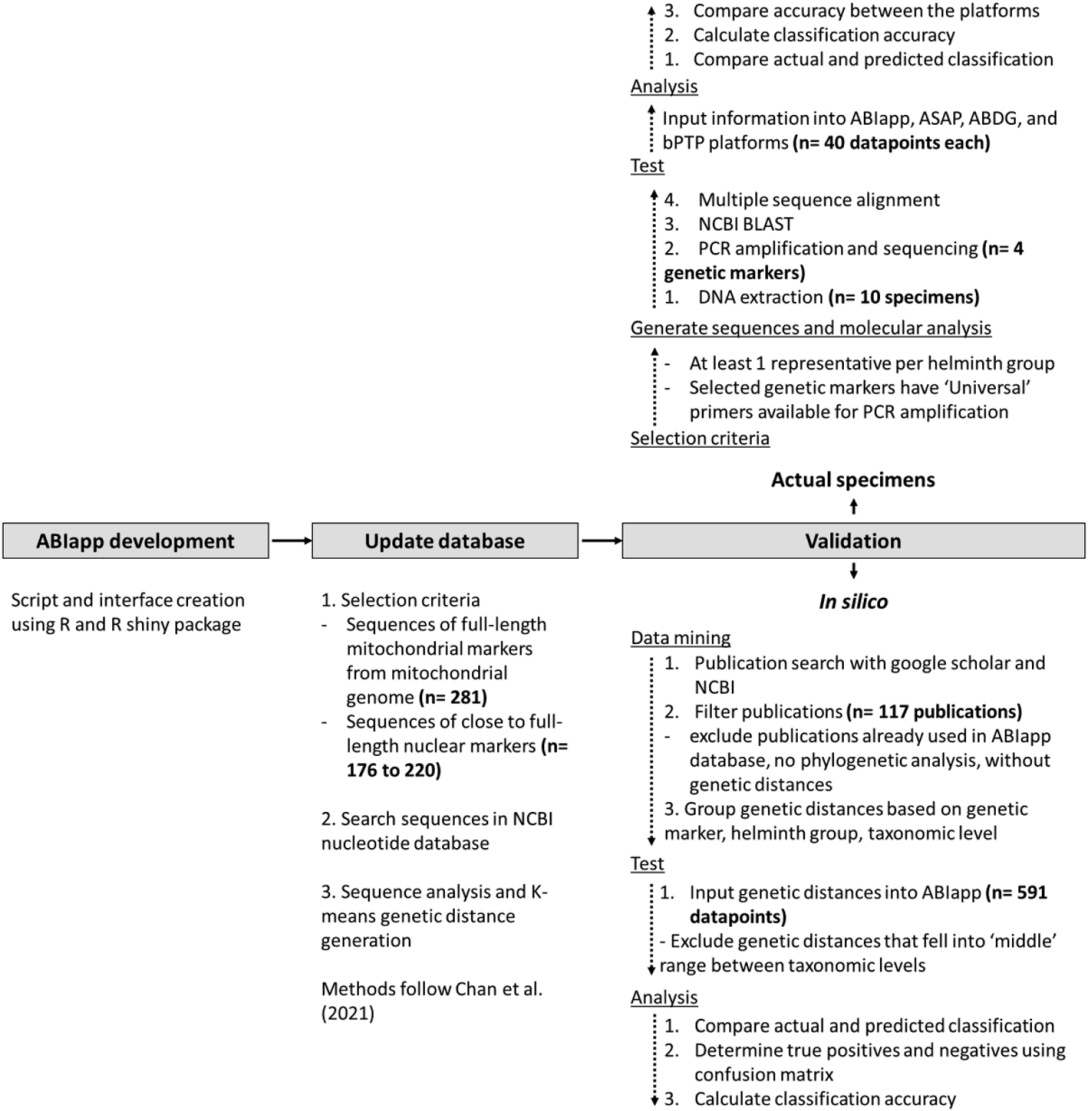
ABIapp validation workflow illustrated in three primary phases: application development, database updating, and validation

A visual representation of the entire validation process for ABIapp is provided in Fig. 3.

## Results and discussion

### Determination of taxonomic boundaries using genetic distances via the K-means algorithm

Spanning ten genetic markers across six helminth groups, estimated genetic distances were generated using the K-means algorithm, where these values were then used as a database and incorporated into ABIapp. The estimated genetic distances generated are in Additional Table 2. Based on the values generated, the taxonomic boundaries (e.g., between species, between genera, between families), as indicated by the maximum and minimum values, vary among helminth groups. For example, the range between species using the *COI* gene for nematode clade I was 6.7 – 17.6%, with a mean of 12.1%, while for trematode Plagiorchiida was 6.6 – 19.1% with a mean of 15.0%, and for cestode was 0.1 – 18.7% with a mean of 15.3%. Similarly, among genetic markers for the same helminth group, the range of genetic distances varied. Thus, as concluded by Chan et al. (2021), utilizing a general value to aid in determining whether specimens are conspecific may be challenging for parasitic helminths, and utilizing ABIapp can be beneficial as a first step to gauge taxonomic boundaries and aid in helminth species identification [10].

### In silico determination of the classification accuracy of ABIapp

**Fig. 4:**
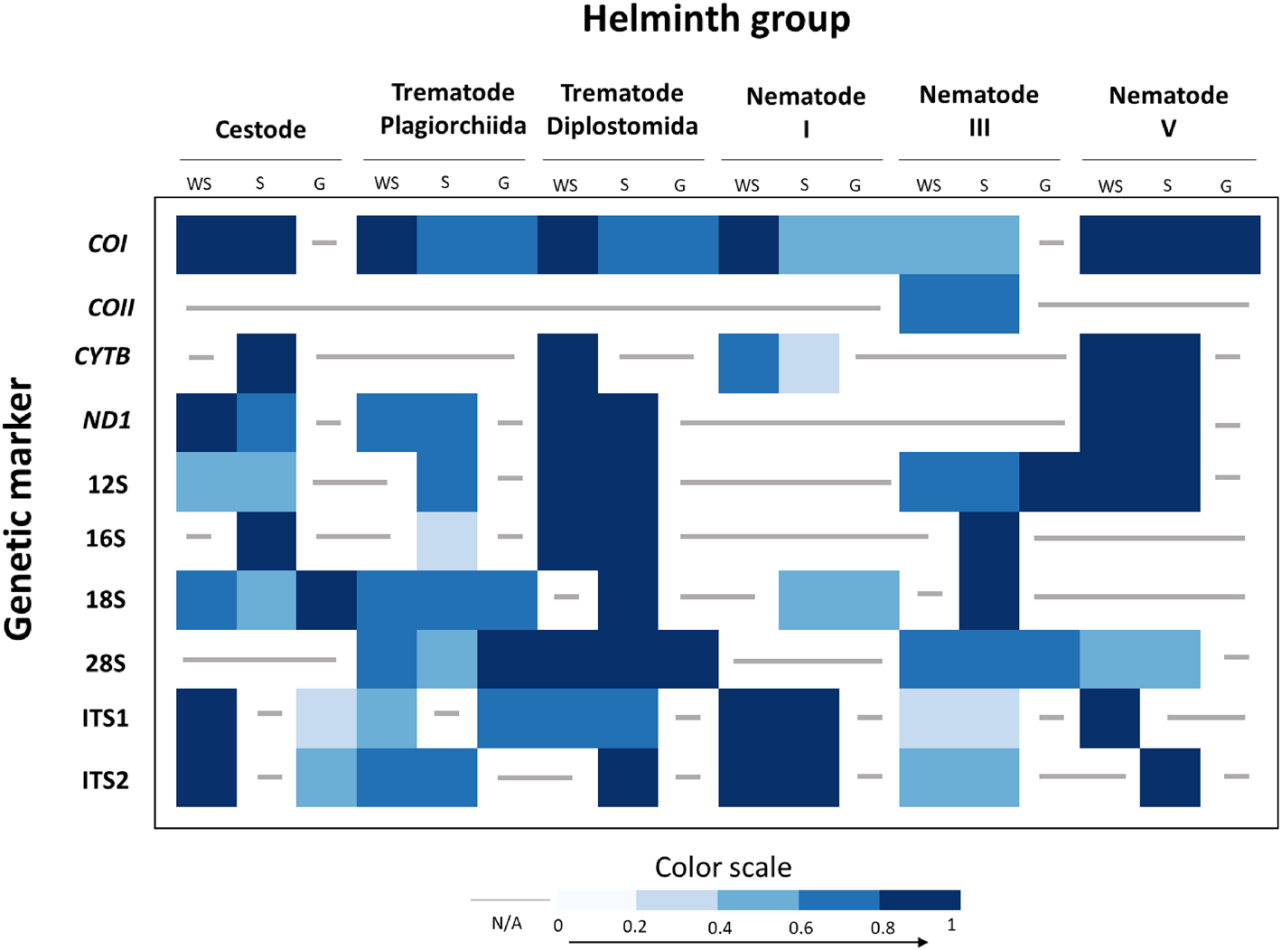
Visualization of ABIapp’s in silico species delimitation classification accuracy. The vertical column displays the ten genetic markers tested, and the horizontal row presents the six helminth groups. Taxonomic levels are denoted as: within species (WS), species (S), and genus (G). The color intensity reflects the percentage of classification accuracy, with gray lines indicating unavailable data (N/A)

With the estimated genetic distances based on the K-means algorithm incorporated into ABIapp, the accuracy of the in silico classification application was investigated. Using 591 genetic distance values across the ten genetic markers for six helminth groups obtained from 117 publications (Additional Table 4), we determined ABIapp’s classification accuracy (Additional Tables 3 and 5, and Additional Fig. 1). Overall, a classification accuracy of 76% was achieved for the ten genetic markers across six helminth groups and three taxonomic hierarchy levels. Figure 4 depicts an intensity map of the classification accuracy for each genetic marker by helminth group at their respective taxonomic levels; darker blue colors indicate higher accuracy. When comparing classification accuracy by helminth group, nematode clade V had the highest at 90%, followed by trematode (Diplostomida) at 89% (Additional Fig. 1a). For the three taxonomic hierarchy levels, the classification accuracy varied slightly, ranging from 74 to 79% (Additional Fig. 1b). Among the genetic markers, the average classification accuracy varied between 68 and 88%, with the mitochondrial *ND1* gene having the highest accuracy and the ITS1 region the lowest (Additional Fig. 1c).

Furthermore, mitochondrial genetic markers demonstrated slightly better classification accuracy than nuclear ones, reinforcing ABIapp’s validity, utility, and accuracy when common mitochondrial genetic markers are used. A potential reason for the lower classification accuracy of nuclear spacer regions might be the presence of repeat regions, which can complicate sequence alignment and genetic distance calculations across different publications [10, 39, 40]. Additionally, as the nuclear 18S and 28S rRNA genes span 4 and 12 domains, respectively, few studies use their complete length. Challenges may arise when different domains are chosen for genetic distance analysis [41].

Our in silico validation underscores ABIapp’s robustness, achieving over 70% accuracy for nine of the ten genetic markers. Furthermore, as the genetic distances tested were not limited to specific groups or helminth hosts, ABIapp is versatile, catering to a wide range of helminth species.

Using the original dataset of cut-off genetic distances from Chan et al. [10], the initial overall classification accuracy from in silico validation was 69% (results not shown). By expanding and updating the database of cut-off genetic distances, incorporating data from about 91 (originally from Chan et al. (2021)) to 281 helminth species in total (the total number of species used for the mitochondrial genes), we enhanced the overall classification accuracy to 76%. This improvement underscores the significance of expanding the number of species to enhance ABIapp’s classification accuracy. Given the growing trend of using molecular genetic markers for helminth identification and the consequent increase in molecular sequences in the NCBI database [2], we plan to update ABIapp’s database of cut-off genetic distance values annually. This will aim to continually refine ABIapp’s classification accuracy and extend its utility for users.

### Classification accuracy with actual specimens

**Fig. 5:**
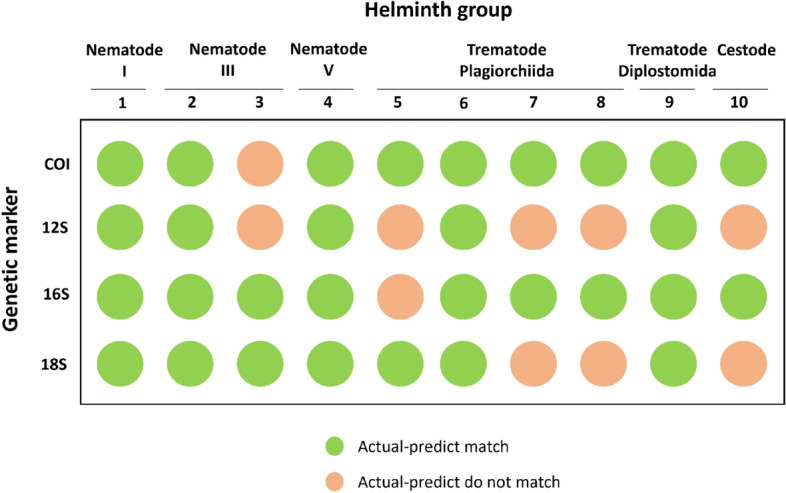
Accuracy of ABIapp classification based on actual specimens. The vertical column displays the four tested genetic markers, while the horizontal row enumerates the ten helminth species as follows: 1- *Trichuris globulosa*, 2- *Gnathostoma* sp., 3- *Tanqua* sp., 4- *Oesophagostomum stephanostomum*, 5- *Gastrothylax* sp., 6- *Eurytrema* sp., 7- *Centrocestus formosanus*, 8- *Stellantchasmus falcatus*, 9- *Clinostomum* sp., 10- *Bertiella* sp.). Green and pink circles indicate matches in classification accuracy

**Fig. 6:**
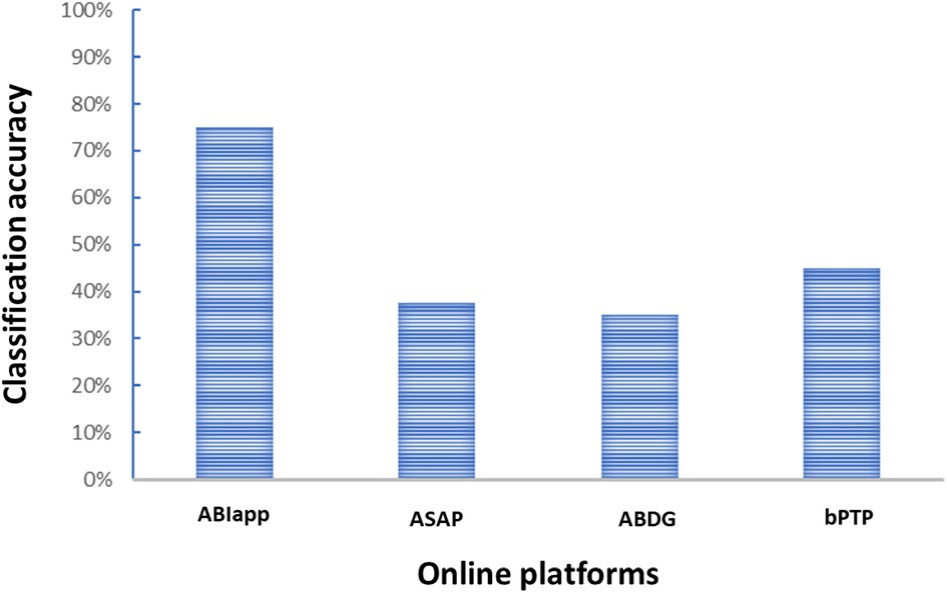
Comparison of ABIapp’s classification accuracy with ASAP, ABDG, and bPTP platforms

From the ten helminth specimens analyzed using the mitochondrial *COI*, 12S, 16S, and nuclear 18S genes, an overall accuracy of 75% was achieved, with 30 out of 40 data points (using 10 specimens with four genetic markers for each specimen) correctly classified. Figure 5 illustrates the classification accuracy for the ten helminth species tested. Moreover, when compared to three online platforms, ABIapp demonstrated superior accuracy. The other platforms registered between 35 and 45% classification accuracy, whereas ABIapp reached 75% (Fig. 6 and Additional Table 6). Among the six helminth groups, cestodes and trematodes (Plagiorchiida) exhibited lower classification accuracy.

In contrast, nematode clade I, V, and trematode (Diplostomida) achieved 100% accuracy. These validation results align with the in silico classification accuracy, where the highest and lowest accuracies were observed for nematode clade V and trematode (Plagiorchiida), respectively. When evaluating the four genetic markers, the mitochondrial *COI* and 16S rRNA genes stood out with a 90% classification accuracy (accurate in nine out of ten data points). This is notable, as the mitochondrial *COI* and 16S rRNA genes are esteemed genetic markers for molecular identification due to their pronounced sequence variation [42, 43].

Discrepancies between actual and predicted results might be ascribed to the number of sequences available in reference databases. For instance, the *COI* gene boasts more reference sequences than the 12S rRNA gene, leading to enhanced classification accuracy when using the *COI* gene in ABIapp. The degree of sequence variation in the genetic marker can also influence results. For instance, with the 18S rRNA gene, even though no sequence variation was detected (between our *S. falcatus* specimen and the reference *S. falcatus*), ABIapp deduced that the sequences came from different species (as indicated in Additional Table 6). ABIapp’s classification inaccuracies became evident when the taxonomic boundaries at the species level, defined using K-means, ranged from 0 to 2.4%. This underscores the importance of employing an auxiliary genetic marker to confirm species, as information from one marker may be insufficient to determine if a species is conspecific [39]. An auxiliary marker should be one from an independent locus (e.g. nuclear and mitochondrial) as despite different degrees of variation, the mitochondrial or nuclear DNA are inherited as a unit. Additionally, the complex taxonomic statuses of certain trematode groups (e.g., within the Opisthorchioidea superfamily) might also lead to inconsistencies [39, 44, 45].

### Benefits of using ABIapp

ABIapp is a convenient application allowing users to visualize helminth species boundaries, making it easy to determine whether a queried sequence is conspecific based on the provided boundaries. By comparing known genetic distances estimated via K-means to the queried taxon, the application offers multiple benefits assisting in accurate species identification, including the potential for delimitation and prospecting.

Firstly, ABIapp has a higher classification accuracy than other available online platforms (75% obtained for ABIapp while the other three platforms ranged from 35 to 45%). The higher accuracy could be due to its use of specific sequence information from a helminth reference database. Although other platforms are available online, ABIapp offers a helminth-specific database, enhancing and increasing the accuracy for species identification.

Secondly, ABIapp incorporates estimated cut-off genetic distances into a web-based application and is also available as an R package, providing users easy access to the tool. The application only requires users to upload a FASTA file (after multiple sequence alignment) or provide a pairwise genetic distance value to determine a specimen’s taxonomic status. With minimal bioinformatics knowledge, users can generate a simple phylogeny, pairwise genetic distance value, and species boundaries of their taxa of interest. Generating genetic distances is straightforward and can be done with freely available bioinformatics software. Since genetic distances are commonly used in publications and DNA barcoding, ABIapp provides an initial gauge on the genetic relatedness of the queried taxon and reference taxon.

Thirdly, the user-friendly interface of ABIapp makes it easy to use. The genetic distance database includes many helminth species found in various hosts and environments, ensuring the application is not restricted to a particular group of helminths. ABIapp can help reduce species misidentification by assessing whether the genetic distance of the queried taxon falls within the interspecies range. Reducing species misidentifications is highly beneficial to advance helminth research, especially since molecular data is easily available on public databases. Utilizing ABIapp as an initial gauge may serve as an important step for subsequent species confirmation. Additionally, by suggesting taxonomic boundaries, the output obtained from ABIapp may aid in species prospecting and delimitation especially in biodiversity surveyswhere many unknown taxa may be present. For instance, if morphological identification suggests conspecific species while genetic distance boundaries suggest otherwise, species prospecting can reveal a possible new species. For species delimitation, ABIapp’s taxonomic boundaries help identify whether the taxon of interest is a distinct species or is part of the same species. Unlike general purpose phylogenetic applications, ABIapp offers the visualization of taxonomic boundaries that are helminth- and genetic marker-specific, providing valuable information that users can utilize.

Finally, by validating the classification accuracy of ABIapp through in silico methods and using actual specimens, we have demonstrated its accuracy and applicability to a broad range of helminth species.

### Assumptions of ABIapp

ABIapp relies on certain assumptions that users should know. First, as only genetic distances or the FASTA file are input into the application, other information, such as morphological characteristics or biological information, is not used to determine a specimen’s taxonomic status. Second, a species’ identity and taxonomic classification were assumed to be correct based on the information provided in the NCBI database. Third, data processing and species identification (e.g., morphology, multiple sequence alignment, genetic distance, and taxa selection) before using ABIapp is subjected to the user’s accuracy. In the case of misidentification, the actual species identity of the queried taxon will not be known as ABIapp can only inform the user about the genetic distance and taxonomic boundary. Lastly, intraspecific variation was assumed to be the lower limit genetic distance value obtained between species, and the species complex status of some helminth species was not accounted for.

### Limitations of the study

Firstly, as the data in ABIapp will be updated yearly, newly uploaded sequences and available species may not be updated for ABIapp yet. Moreover, as helminths exhibit extensive genetic diversity, a larger dataset may be beneficial to enhance the classification accuracy of ABIapp. Also, as the data in the current version of ABIapp focused on animal and human parasitic helminths from clades I (Trichocephalida), III (Spirurida), and V (Strongylida), the application cannot be used for plant parasitic helminths (in clades II and IV). Increasing the dataset may be included in future updates. Secondly, as genetic distances are used as a basis for ABIapp, evolutionary inferences should be avoided. However, the results obtained from the application can serve as a gauge to get an idea of the genetic relatedness of the queried specimen to reference taxa. Thirdly, genetic distances were obtained from other publications for the in silico validation; however, the various methods used in these studies to generate genetic distances were not accounted for. Fourthly, ABIapp’s classification accuracy ranges between 75 and 76%, demonstrating its high efficiency as a pioneering program for parasitic helminths, though there is still room for improvement. However, compared with other online tools, the classification accuracy of ABIapp for helminths (nematodes, trematodes, and cestodes) is superior, thus serving as a promising application for helminth researchers. Finally, other machine learning algorithms were not compared and cross-validated with the K-means clustering algorithm used for ABIapp. Also, confidence intervals for the classification accuracy of ABIapp were not calculated due to the nature of K-means clustering. The lack of comparisons may compromise the classification accurary of ABIapp.

In conclusion, we have developed a convenient and user-friendly application that applies to a broad audience to assess helminth species boundaries which serves as a preliminary tool and can eventually aid users in making informed choices regarding species identification. The robustness of ABIapp for determining helminth taxonomic boundaries was also validated for its classification accuracy via in silico methods and the use of actual specimens. The database of genetic distances for ABIapp will be updated annually to keep up to date with the increasing number of sequences available in molecular databases. ABIapp represents a new frontier for helminth taxonomy that is now readily available for researchers in helminthology.

## Supplementary Information

Additional file1 (DOCX 88 KB)

Additional file2 (XLSX 69 KB)

Additional file3 (XLSX 20 KB)

Additional file4 (XLSX 22 KB)

Additional file5 (XLSX 29 KB)

Additional file6 (XLSX 43 KB)

Additional file7 (XLSX 19 KB)

## Data Availability

The datasets supporting the conclusions of this article are included within the article, its additional files, and the source code is available at https://github.com/slphyx/ABIApp. The sequences generated in this study are in the NCBI database under accession numbers PP066032 – PP066041 for *COI*, PP077008 – PP077017 for 12S, PP077018 – PP077027 for 16S, and PP077028 – PP077037 for 18S. Project name: Applying Taxonomic Boundaries for Species Identification of Helminths (ABIapp). Project home page: https://moru.shinyapps.io/ABIapp/. Operating system(s): Platform independent. Programming language: R. Other requirements: None. License: GNU GPL. Any restrictions to use by non-academics: None
