# Supplementary material for: Validating a web application’s use of genetic distance to determine helminth species boundaries and aid in identification
Source: BMC Bioinformatics. 2025 Mar 18;26:85. doi: 10.1186/s12859-025-06098-0 (PMC11917154; doi:10.1186/s12859-025-06098-0)

**Additional figures 1a – 1c: Average classification accuracy based on *in silico* validation**

**Additional figure 1a: Average classification accuracy per helminth group with *in silico* validation**


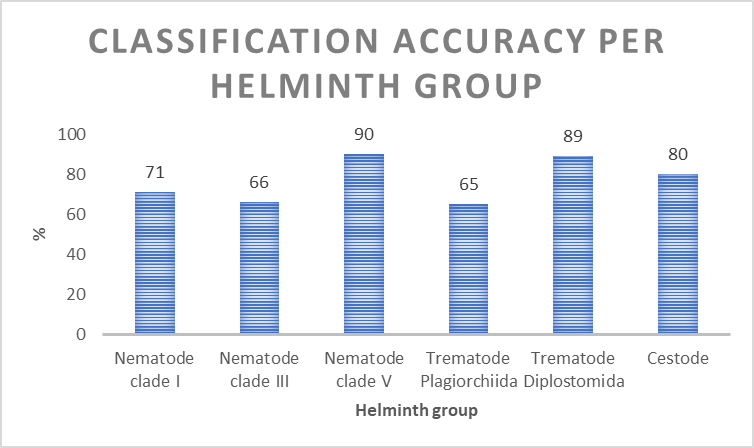


**Additional figure 1b: Average classification accuracy per taxonomic level with *in silico* validation**


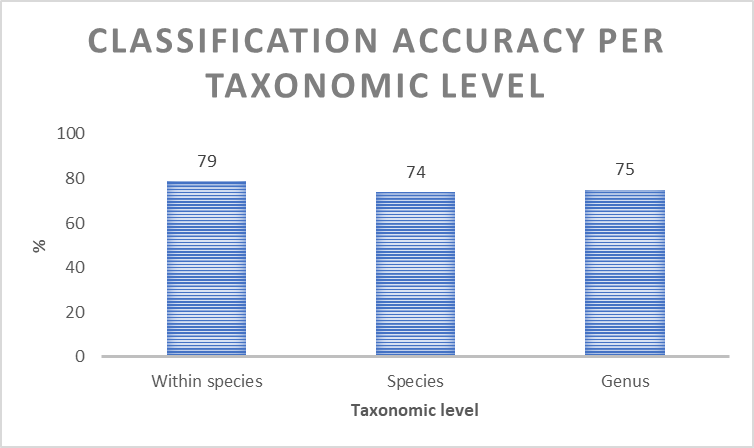


**Additional figure 1c: Average classification accuracy per genetic marker with *in silico* validation**


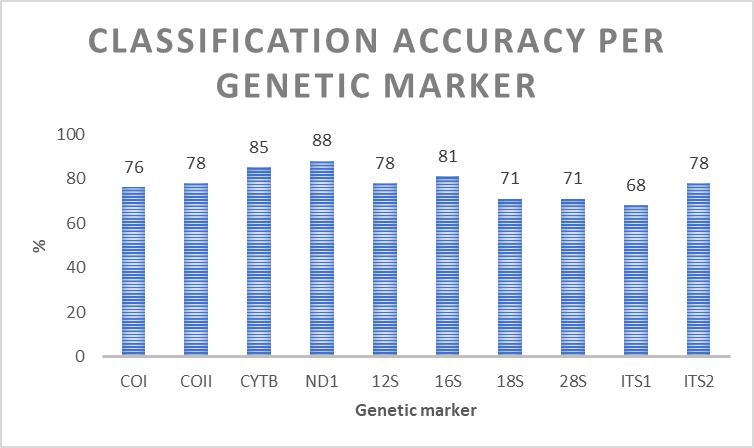

Supplement: Supplementary file 1 — Additional file1 (DOCX 88 KB) [file 12859_2025_6098_MOESM1_ESM.docx]
